# N-glycosylation Dictates Proper Processing of Organic Anion Transporting Polypeptide 1B1

**DOI:** 10.1371/journal.pone.0052563

**Published:** 2012-12-20

**Authors:** Juan Yao, Weifang Hong, Jiujiu Huang, Kai Zhan, Hong Huang, Mei Hong

**Affiliations:** 1 College of Life Science, South China Agricultural University, Guangzhou, China; 2 School of Information, University of South Florida, Tampa, Florida, United States of America; University of Cambridge, United Kingdom

## Abstract

Organic anion transporting polypeptides (OATPs) have been extensively recognized as key determinants of absorption, distribution, metabolism and excretion (ADME) of various drugs, xenobiotics and toxins. Putative N-glycosylation sites located in the extracellular loops 2 and 5 is considered a common feature of all OATPs and some members have been demonstrated to be glycosylated proteins. However, experimental evidence is still lacking on how such a post-translational modification affect the transport activity of OATPs and which of the putative glycosylation sites are utilized in these transporter proteins. In the present study, we substituted asparagine residues that are possibly involved in N-glycosylation with glutamine residues and identified three glycosylation sites (Asn134, Asn503 and Asn516) within the structure of OATP1B1, an OATP member that is mainly expressed in the human liver. Our results showed that Asn134 and Asn516 are used for glycosylation under normal conditions; however, when Asn134 was mutagenized, an additional asparagine at position 503 is involved in the glycosylation process. Simultaneously replacement of all three asparagines with glutamines led to significantly reduced protein level as well as loss of transport activity. Further studies revealed that glycosylation affected stability of the transporter protein and the unglycosylated mutant was retained within endoplasmic reticulum.

## Introduction

The organic anion-transporting polypeptides (OATPs, gene symbol *SLCO*) are a family of transporters that mediate sodium-independent transport of a wide spectrum of structurally independent compounds [1]. Substrates of OATPs are mainly amphipathic organic molecules including bile salts, hormones and their conjugates, toxins and different drugs. In recent years, OATPs have been extensively recognized as key determinants of absorption, distribution, metabolism and excretion (ADME) of various drugs, xenobiotics and toxins because of their broad substrate specificity, wide tissue distribution and the involvement of drug-drug interaction [2], [3]. So far there are 12 members of the human OATP family: OATP1A2, 1B1, 1B3, 1B7, 1C1, 2A1, 2B1, 3A1, 4A1, 4C1, 5A1 and 6A1 [4]–[7]. Some OATPs are expressed ubiquitously; while others, such as OATP1B1 and OATP1B3, are predominantly found in certain organs or tissues. OATP1B1 is mainly located at the basolateral membrane of human hepatocytes and play an essential role in drug clearance from the body [8]. In recent years, more and more drugs are identified as substrates of this OATP family member [9].

As the most common and diverse form of post-translational modification for newly synthesized proteins, glycosylation is widely found in integral membrane proteins of higher organism and also common in secretory proteins [10]. Many transporters are found to possess glycosylation sites in their amino acid sequences. For instance, one common feature for the organic anion transporter (OAT) family members is the presence of consensus sites for N-linked glycosylation within the extracellular loop between transmembrane domain (TM) 1 and 2. Disruption of all four putative glycosylation sites in both mOAT1 and hOAT1 impaired the trafficking of the transporter to the cell surface as well as the transport of *p*-amminohippuric acid [11]. Study on glycosylation of organic cation transporter (OCT) showed that the oligosaccharide moiety(s) may be located close to the binding site for organic cations and that glycosylation of OCT is critical for the proper conformation of the binding pocket [12]. Putative N-glycosylation sites (N*X*(S/T), where X is any amino acid except proline) located in the extracellular loops 2 and 5 is considered a common feature of all OATP proteins [6]. Wang et al. showed that rat oatp1a1 is glycosylated at asparagines 124, 135, and 492 in the second and fifth extracellular loops, while the potential glycosylation site at asparagine 62 is not utilized [13]. Although it has been demonstrated that some OATPs are glycosylated proteins [14]–[16], experimental evidence is still lacking on how such a post-translational modification affects the function of OATPs and which of the putative glycosylation sites are utilized in these transporter proteins.

In the present study, we disrupted the putative N-glycosylation sites (Asn134, 432, 503, 516 and 617) in OATP1B1 by substituting asparagine (N) with glutamine (Q) and assessed these mutant transporters in HEK293 cells. Our results revealed that asparagine 134, 516 and 503 are glycosylated in OATP1B1. When all three sites were simultaneously substituted with glutamine, the transporter protein became unglycosylated and presented a dramatically decreased level of protein expression as well as loss of transport function.

## Materials and Methods

### Materials

[^3^H]Estrone-3-sulfate (E-3-S) was purchased from PerkinElmer Life Sciences (Waltham, MA). Sulfosuccinimidyl 2-(biotinamido)-ethyl-1, 3-dithiopropionate (NHS-SS-biotin) and streptavidin-agarose beads were from Thermo Scientific (Rockford, IL). All other reagents were purchased from Sigma except where otherwise stated.

### Site-directed Mutagenesis

Mutant transporters were generated using QuikChange Lightning Site-Directed Mutagenesis Kit from Agilent (Santa Clara, CA). The pReceriver M07 vector containing the *SLCO1B1* cDNA and 3-HA tags at the C-terminus was obtained from Genecopoeia (Rockville, MD) and used as the template for the mutagenesis. All mutant sequences were confirmed by the dideoxy chain termination method.

### Cell Culture and Transfection of Plasmid Constructs into Cells

HEK293 cells (ATCC, Manassas, VA) were grown at 37°C and 5% CO_2_ in Dulbecco’s modified Eagle’s medium (Invitrogen, Carlsbad, CA) supplemented with 10% fetal bovine serum (Invitrogen). Confluent cells in 48-well or 6-well plates were transfected with DNA plasmid using LipofectAMINE 2000 reagent (Invitrogen) following manufacturer’s instruction. Transfected cells were incubated for 48 h at 37°C and then used for transport assay and cell surface biotinylation.

### Cell Surface Biotinylation and Western Blot

Cell surface expression level of OATP1B1 and their mutants were examined using the membrane-impermeable biotinylation reagent NHS-SS-biotin with the method described previously [17]. Briefly, forty-eight hours after transfection, HEK293 cells in 6-well plates were labeled with NHS-SS-biotin (0.5 mg/ml in phosphate-buffered saline (PBS)). The cells were then dissolved on ice for 1 h in 400 μl of RIPA buffer (50 mM Tris, 150 mM NaCl, 0.1% SDS, 1% NP-40, protease inhibitors phenylmethylsulfonyl fluoride, 200 μg/ml, leupeptin, 3 μg/ml, pH 7.4). The cell debris was removed by centrifugation and streptavidin-agarose beads were added to bind the biotin-labeled cell membrane proteins. Protein was released and denatured in 4×Laemmli buffer and loaded onto a 7.5% SDS-polyacrylamide electrophoresis gel and then transferred electrophoretically to a polyvinylidene difluoride membrane (Millipore, Billerica, MA). OATP1B1 was detected with an anti-HA antibody (Cell Signaling Technology, Danvers, MA).

### Deglycosylation of Plasma Membrane Proteins with N-glycosidase F

Cell surface proteins were isolated with cell surface biotinylation method described above. Proteins were then denatured in 0.5%SDS and 1% β-mercaptoethanol at 75°C for 15 min. The denatured proteins were cooled to room temperature, then incubated with 50 mM sodium hydrogen phosphate (pH7.5), 1% Nonidet P-40 and 5 μl of N-glycosidase F (100 units/μl, Roche, Indianapolis, IN) overnight at 37°C.

### Uptake Assay

Cells in 48-well plates were used for transport measurement as described before [17] with minor modifications. Briefly, cells were incubated with uptake solution (125 mM NaCl, 4.8 mM KCl, 5.6 mM D-glucose, 1.2 mM KH_2_PO_4_, 25 mM HEPES, 1.2 mM CaCl_2_, and 1.2 mM MgCl_2_, pH7.4 and [^3^H]E-3-S) for 2 min at 37°C and the uptake process was stopped by adding ice-cold PBS solution into each well. Cells were then solubilized in 0.2 N NaOH and the radioactivity of cell lysate was measured with a liquid scintillation counter Triathler-Hidex (Hidex, Finland). Uptake count was standardized by the amount of protein in each well.

### Immunofluorescence Analysis of Transfected Cells

Forty-eight hours after transfection, transfected HEK293 cells were washed three times with PBS and fixed with 4% paraformaldehyde for 20 min at room temperature. The fixed cells were permeabilized with 0.1% Triton X-100 for 10 min, washed with PBS and incubated with PBS containing 5% goat serum for 1 h at room temperature. Primary antibodies (dilution 1∶100) in the same medium was then added and incubated overnight at 4°C. The cells were washed, and bound primary antibodies were detected by reaction with Alexa Fluor 488 goat anti-mouse or Alexa Fluor 555 goat anti-rabbit IgG antibody diluted 1∶1000 (Cell Signaling Technology) for 1 h. Cells were thoroughly washed and the cover glasses were mounted in Fluoromount mounting medium. Samples were examined using a Zeiss LSM 710 confocal microscope (Carl Zeiss, Oberkochen, Germany).

### Statistical Analysis

Data statistical analysis was carried out using Student’s *t*-test. Differences between means are regarded as significant if *p*<0.05.

## Results

### N-Glycosylation of OATP1B1

**Figure 1:**
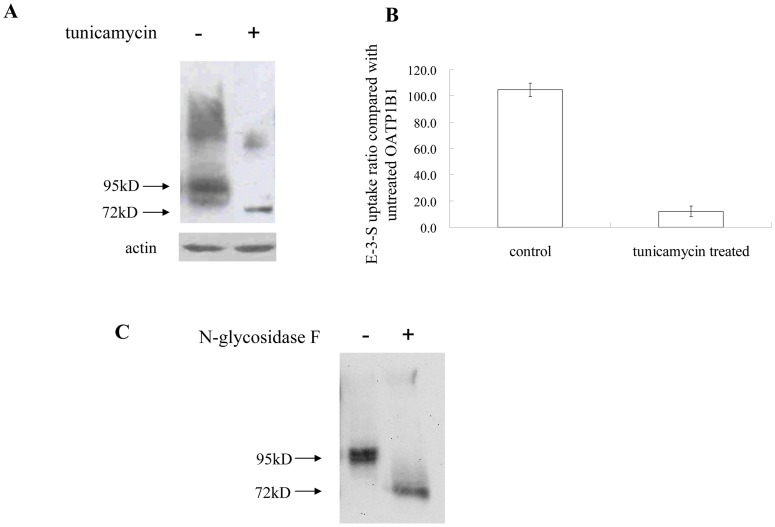
OATP1B1 is glycosylated in HEK293 cells. A. The effect of tunicamycin on protein size of OATP1B1. B. Transport of E-3-S (0.1 μM) in OATP1B1-expressed HEK293 cells with or without tunicamycin treatment. C. Plasma membrane proteins from cells expressing OATP1B1 treated with N-glycosidase F. Cells expressing OATP1B1 were treated with 0.5 μg/ml of tunicamycin for 42 h before analysis. For protein expression, cells were lysed with RIPA buffer, separated by SDS-PAGE, followed by Western blotting with anti-HA antibody. Fifty micrograms of protein was loaded for each lane. Transport function of tunicamycin treated cells was expressed as a percentage of the uptake measured in the untreated control. The results represent data from three experiments, with triplicate measurements for each sample. The results shown are means ± S.E. (*n  = *3). For glycosidase treatment, cell surface proteins were biotinylated and precipitated with streptavidin beads. Proteins were then denatured with 0.5%SDS and 1% β-mercaptoethanol at 75°C for 15 min. The denatured proteins were incubated with or without N-glycosidase F overnight at 37°C before subjected to SDS-PAGE.

It has been shown that mature OATPs are glycosylated proteins *in vivo* [14]–[16]. To investigate whether OATP1B1 is glycosylated and whether such a post-translational modification affects transport activity in HEK293 cells, we first treated cells expressing OATP1B1 with tunicamycin, a general inhibitor of glycosylation. Treatment of 0.5 μg/ml of tunicamycin for 42 h resulted in a reduced molecular mass in Western blot analysis. The molecular size of the signal was ∼72 kDa and consistent with the predicted size (unglycosylated form) for OATP1B1 (Fig. 1A). Moreover, the pretreatment of tunicamycin significantly suppressed transport activity as well as protein level of OATP1B1 (Fig.1A&B). We also treated plasma membrane proteins isolated from cells expressing OATP1B1 with N-glycosidase F, an enzyme that removes the N-linked carbohydrate groups from glycoproteins. As indicated in Figure 1C, the molecular mass reduced from 95 kDa to a smaller size of 72 kDa. These results suggested that OATP1B1 is glycosylated in HEK293 cells and that glycosylation affects its transport function.

### Effect of Disruption of Individual Putative Glycosylation Sites

**Figure 2:**
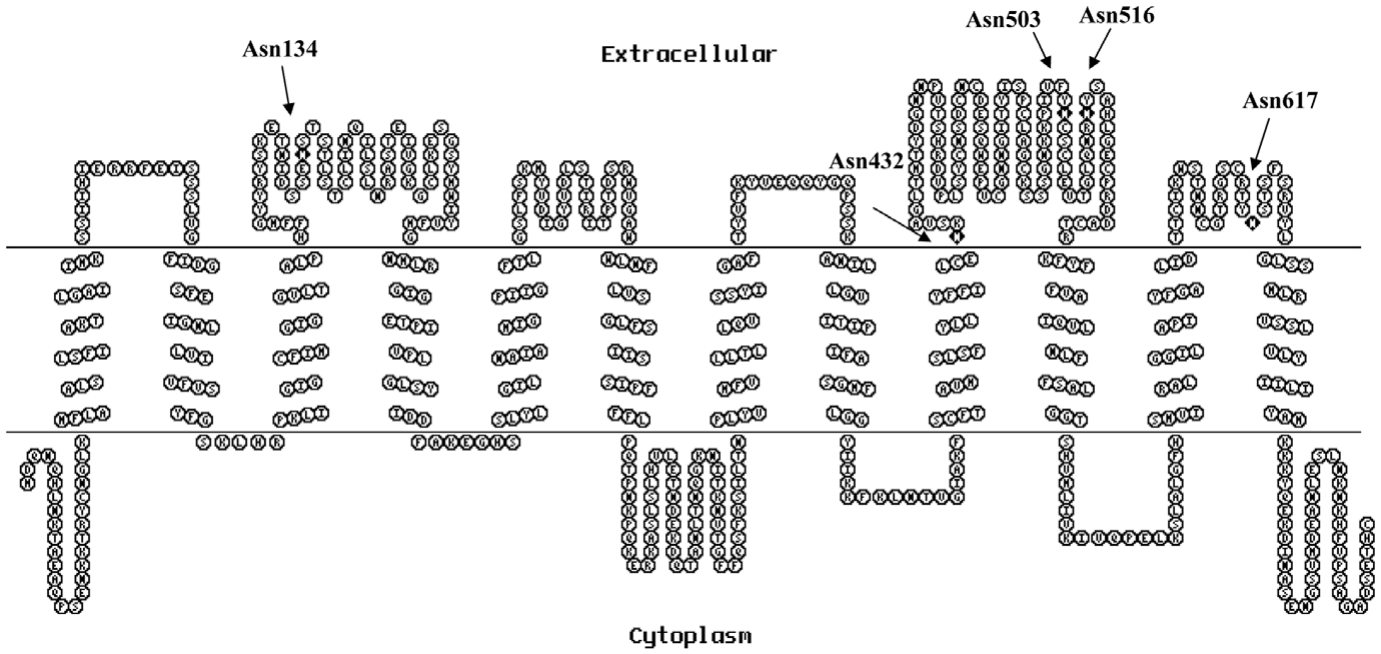
Secondary structure model of OATP1B1. Putative glycosylation sites were identified with NetNGlyc 1.0 Server and compared with membrane protein topology prediction analysis TopPred (Kyte-Doolittle hydrophobicity scale), only asparagines located extracellularly were considered as candidates for glycosylation sites. Putative sites were marked as black diamonds and each position was indicated with arrows.

**Figure 3:**
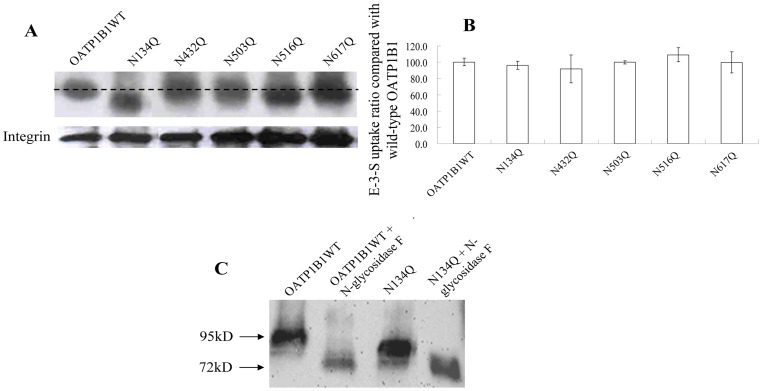
Effect of single disruption of putative glycosylation sites. A. Western blot analysis of single mutants. Plasma membrane proteins were isolated from cells expressing wild-type OATP1B1 and mutants through biotinylation. Same blot was probed with integrin antibody as surface protein loading control. B. Transport of E-3-S (0.1 μM) in OATP1B1 and five single mutants. Transport function of mutants was expressed as a percentage of the uptake measured in wild-type. The results represent data from three experiments, with triplicate measurements for each mutant. The results shown are means ± S.E. (*n  = *3). C. Plasma membrane proteins of wild-type OATP1B1 and N134Q treated with N-glycosidase F. Glycosidase treatment was performed as described above.

OATP1B1 has a proposed topology of 12 transmembrane domains with both amino- and carboxyl- termini located intracellularly. According to computer analysis with Kyte- Doolittle hydrophobicity scale, five consensus sites (N*X*(S/T)) for N-glycosylation, located in the second, fifth and sixth extracellular loops of OATP1B1, were identified (Fig. 2). To assess which of these positions are utilized for glycosylation, we disrupted the glycosylation sites individually by site-directed mutagenesis, substituting asparagine (N) with glutamine (Q). As shown in Figure 3, the replacement of N432, N503 and N617 with glutamine did not seem to affect the size of the transporter protein. On the other hand, mutant N134Q presented a much reduced molecular weight compared with that of the wild-type OATP1B1. Mutation on position 516 also led to decrease in molecular weight, though to a much lesser extend than N134Q. However, all five mutants retained cell surface protein expression level as well as transport activity (Fig.3A&B). Since the size change in N516Q was subtle, we treated N134Q mutant with N-glycosidase F to make sure whether position 134 is the only glycosylation site of OATP1B1 and responsible for the increased molecular weight of the mature transporter protein. Such a treatment resulted in a more reduced molecular mass (Fig.3C) compared with the untreated sample, implicating that in addition to Asn134, there are other positions involved in glycosylation.

### Effect of Multiple Disruptions of Putative Glycosylation Sites

**Figure 4:**
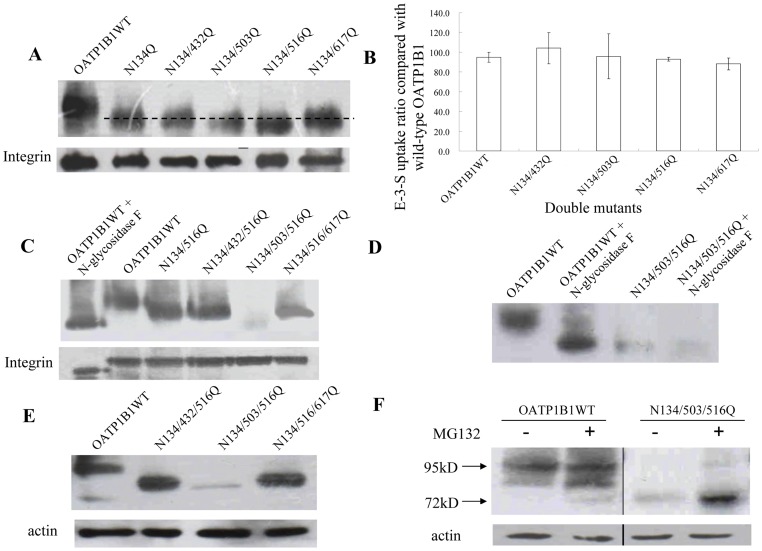
Effect of multiple disruption of OATP1B1 glycosylation sites. A. Western blot analysis for plasma membrane protein expression of double mutants. B. Transport of E-3-S (0.1 μM) in OATP1B1 and double mutants. C. Western blot analysis for plasma membrane protein expression of triple mutants. D. Plasma membrane proteins of wild-type OATP1B1 and N134/503/516Q treated with N-glycosidase F. E. Total protein expression of OATP1B1 and triple mutants. Same blot was probed with actin antibody as loading control. F. Western blot analysis of wild-type OATP1B1 and triple mutant N134/503/516Q treated with proteasome inhibitor MG132. Cells were treated with 10 μM MG132 for 6 h before being lysed with RIPA buffer and subjected to Western blotting. Fifty micrograms of protein was loaded for each lane.

**Figure 5:**
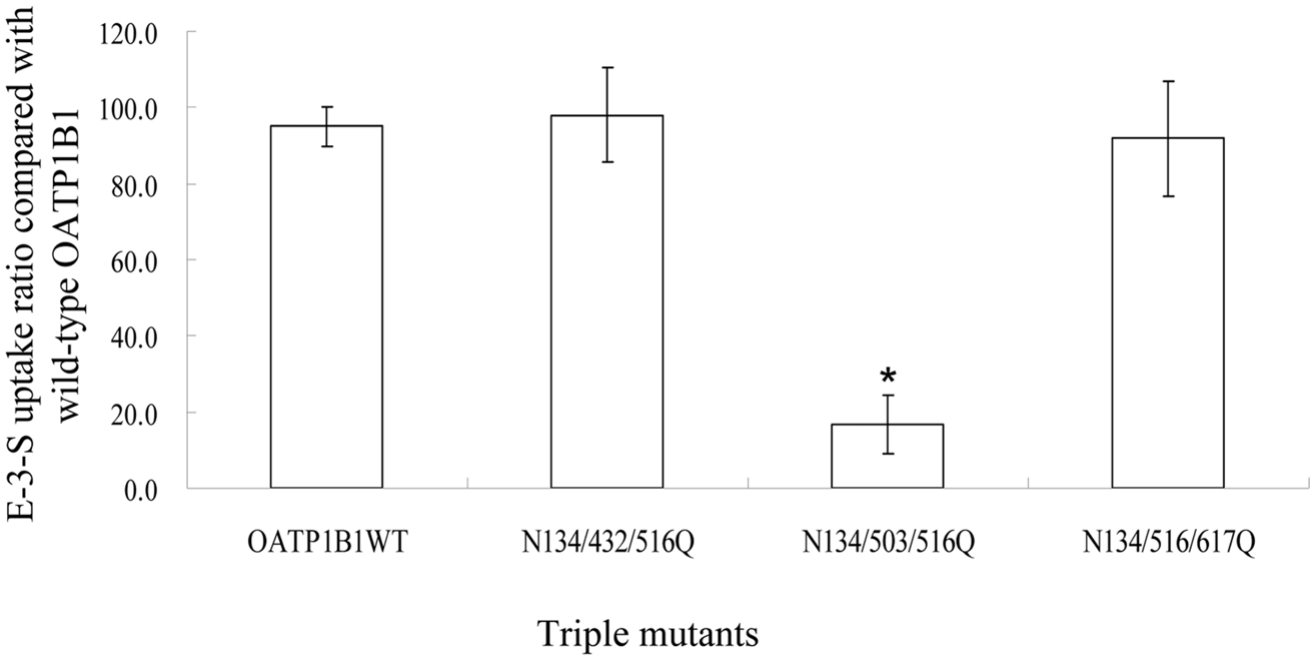
Functional analysis of triple glycosylation site mutants. Cells were incubated with 0.1 μM E-3-S for 2 min at 37°C. Transport function of mutants was expressed as a percentage of the uptake measured in wild-type OATP1B1. The results represent data from three experiments, with triplicate measurements for each mutant. The results shown are means ± S.E. (*n  = *3). Asterisks indicate values significantly different (*p*<0.05) from that of wild-type OATP1B1.

To further investigate the participation of other putative sites in glycosylation, we then generated double mutants using N134Q as the template. Again, cell surface expression level and transport activity of all four double mutants were comparable with that of the wild-type OATP1B1, though molecular weight of double mutant N134/503Q and N134/516Q both decreased marginally (Fig. 4A&B) compared to that of N134Q. Since the single substitution of asparagine with glutamine at position 134 and 516 both led to a reduced molecular mass, suggesting the two positions are utilized under normal conditions, triple mutants using N134/516Q as template were generated. As shown in Figure 4C, simultaneously substituting Asn134, 503 and 516 with glutamine caused significantly decreased protein level on plasma membrane(∼10%). The triple mutant had a smaller molecular weight that was similar to the 72 kDa unglycosylated form of OATP1B1. To confirm that there are no additional sites for N-glycosylation in OATP1B1, we treated cells expressing the triple mutant with N-glycosidase F. As shown in Fig. 4D, molecular mass of N134/503/516Q was the same with or without glycosidase treatment, indicating these three locations are the only glycosylation sites within the structure of OATP1B1. To investigate whether the level of N134/503/516Q on the plasma membrane was reduced due to a decreased amount of total protein present in the cell or the failure of transporter protein targeting to the cell surface, we analyzed total protein level of the triple mutant. Our results showed that total protein level of N134/503/516Q was also greatly reduced, correlated well with its cell surface expression, suggesting the absence of glycosylation in OATP1B1 affected total protein level (Fig. 4E). Moreover, treatment with MG132, a specific and potent proteasome inhibitor, resulted in accumulation of the 72 kDa unglycosylated transporter protein in total cell extracts of both the wild type and N134/503/516Q mutant (Fig. 4F). In addition, the impact of simultaneously mutagenized these three putative glycosylatoin sites on the function of OATP1B1 was examined. Consistent with our Western blot analysis, the triple mutant showed much reduced transport activity (Fig. 5).

### N134/503/516Q was Retained within the Endoplasmic Reticulum

**Figure 6:**
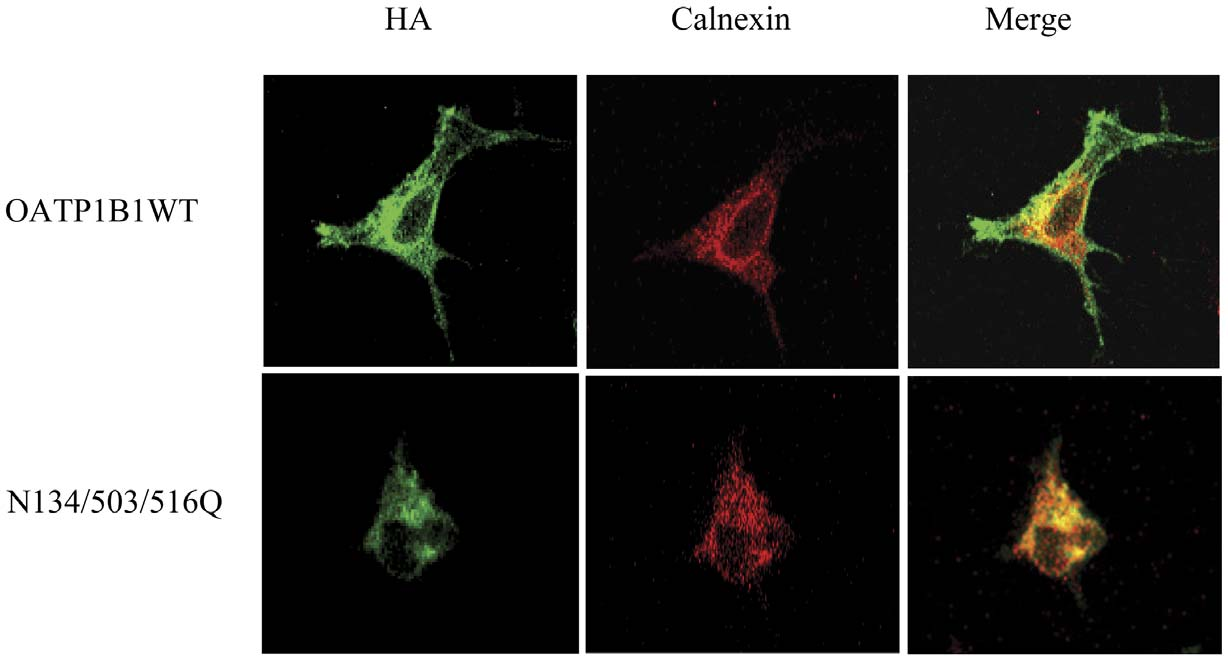
Immunofluorescence study of triple mutant N134/503/516Q. Cells expressing OATP1B1 wild-type and N134/503/516Q were stained with anti-HA antibody (1∶100) and anti-calnexin antibody (1∶100) and reacted with Alexa Fluor 488 goat anti-mouse IgG or Alexa Fluor 555 goat anti-rabbit IgG antibody. Specific immunostaining shown as green (OATP1B1) or red (calnexin) fluorescence.

Since N134/503/516Q had a much reduced protein level and responded to proteasome inhibitor, we next wanted to identify the subcellular location of the mutant so as to have a better understanding of the degradation process. As shown in Figure 6, immunofluorescence signal of N134/503/516Q was colocalized with the endoplasmic reticulum (ER)-specific protein calnexin, suggesting the triple mutant was trapped in the protein processing organelle. On the other hand, the wild-type transporter protein was located on the cell membrane as well as in the ER.

## Discussion

Glycosylation is a major post-translational modification in membrane proteins. Studies had shown that such a process can impair membrane targeting as well as protein stability [11], [13], [18]. Moreover, naturally occurring glycosylation-defective mutant had been observed in organic anion transporting polypeptide family member OATP1A2 [15], suggesting this post-translational modification may be essential for proper function of various transporter proteins.

In the present study, we investigated the possible sites involved in glycosylation of OATP1B1 and how such a modification affects the function of the transporter protein. According to the computer model based on Kyte-Doolittle hydrophobicity scale, OATP1B1 has 12 putative transmembrane domains. There are five consensus sites (N*X*(S/T)) for N-glycosylation located in the second, fifth and sixth extracellular loops of OATP1B1 and possibly involved in its glycosylation process. Site-directed mutagenesis revealed that there was a size reduction occurred in N134Q and N516Q (Fig. 3A). However, such a replacement of individual asparagine residues affected neither cell surface protein level nor transport activity of the mutants. Interestingly, for double mutants that used N134Q as the template, N134/503Q showed a similar protein molecular size shift as N134/516Q did, suggesting that an additional site at Asn503 is utilized when Asn134 was mutagenized. However, it is also possible that Asn503 is used for glycosylation under normal conditions though no size change was observed. The single mutation at this position might not cause a size reduction as obvious as N134Q and N516Q. Again, simultaneous mutation of any two of these three asparagine residues only affected size of the transporter protein. Cell surface protein level and transport function, on the other hand, were unchanged. Only when all three sites of Asn134, 503 and 516 were eliminated would it lead to dramatically reduced protein level on the plasma membrane as well as its uptake function. Further analysis demonstrated that the decreased level of transporter protein on cell surface was due to the reduced total protein expression, implicating glycosylation may play a role in maintaining protein stability. Indeed, treatment with MG132, a potent proteasome inhibitor, resulted in partial recovery of the 72 kDa unglycosylated form of the transporter protein (Fig. 4F). That stability of OATP1B1 may be affected by glycosylation was also evidenced in tunicamycin treatment. HEK293 cells expressing wild-type OATP1B1 treated with tunicamycin showed not only lower molecular weight but also much reduced protein level compared with the untreated control (Fig. 1A). Therefore, the much decreased transport function of OATP1B1 triple mutant was likely due to the significantly reduced level of protein expression. A previous study on rat oatp1a1 demonstrated that three (Asn124, 135, and 492) out of the four putative glycosylation sites in oatp1a1 are utilized and that the fully unglycosylated transporter protein only had limited transport function due to its reduced cell surface expression. However, unlike what was observed in our current study, total protein level of the unglycosylated oatp1a1 was almost unchanged [13], suggesting glycosylation plays different roles in different transporter proteins. It is interesting to note that though the glycosylation sites identified in oatp1a1 is not a one-to-one pair-up with the three asparagine residues in OATP1B1, they both are located on extracellular loop 2 or 5 of the transporter protein structure. Putative glycosylation sites in other positions such as Asn617 localized in extracellular loop 6 seems not to be involved in the glycosylation process. Polymorphism analysis of *SLCO1A2* gene that encodes another OATP member OATP1A2 revealed a genetic variation, A404T, which leads to substitution of an asparagine residue at position 135 with an isoleucine. This variant showed an apparent shift in the molecular size of the transporter as well as a much reduced protein level, implicating that the mutation at a single glycosylation site of OATP1A2 could affect stability of the protein [15]. Although Asn135 in OATP1A2 corresponds to Asn134 in OATP1B1, our results demonstrated that a single replacement of asparagine residue with glutamine had no effect on either protein level or transport activity of OATP1B1. Whether this is due to the difference between OATP family members, or the substitution of asparagine with isoleucine resulted in a more dramatic effect on the protein than a structurally similar glutamine remains to be elucidated.

It has been shown that oligosaccharide chains confer stability on many extracellular glycoproteins. Some proteins require *N*-linked oligosaccharides in order to fold properly in the ER [19]. Moreover, glycosylation is often cited as having a stabilizing effect upon proteins with respect to proteolysis [20]. The initial addition of oligosaccharide occurs in the ER. Unglycosylate proteins are often considered as misfolded, remained in the ER by default and eventually subjected to degradation [21]. Our immunocytochemical analysis demonstrated that the unglycosylated mutant N134/503/516Q was retained within the ER, implicating that for OATP1B1, glycosylation may be required for its proper folding that ensures its exit from the ER. The fully unglysylated form (N134/503/516Q) could not pass the quality control machinery, and thus sorted as misfolded proteins and targeted for degradation.

In summary, our study identified three glycosylation sites, i.e. Asn134, Asn503 and Asn516, which located at extracellular loop 2 and 5 of OATP1B1; while Asn432 and Asn617, the other two putative sites, do not seem to participate in glycosylation process of the transporter protein. Disruption of individual glycosylation site did not affect proper processing and transport function of OATP1B1. On the other hand, simultaneously mutation of all three glycosylation sites resulted in a significantly reduced total protein level, which led to loss of uptake function. The unglycosylated form of the transporter was retained within the ER and may be subjected to accelerated degradation.
